# Extraneural hemangioblastoma of the kidney: the challenge for clinicopathological diagnosis

**DOI:** 10.1136/jclinpath-2015-202900

**Published:** 2015-07-22

**Authors:** Yong Wu, Tao Wang, Pei-Pei Zhang, Xiaoqun Yang, Jian Wang, Chao-Fu Wang

**Affiliations:** 1 Department of Pathology, Fudan University Shanghai Cancer Center, Shanghai, China; 2 Department of Oncology, Shanghai Medical College, Fudan University, Shanghai, China

**Keywords:** KIDNEY, RENAL CANCER, IMMUNOHISTOCHEMISTRY

## Abstract

**Background:**

Hemangioblastoma is a benign cerebellar tumour which may occur as a sporadic entity or in association with von Hippel-Lindau (VHL) disease in approximately 25% of cases. Renal hemangioblastoma (RH) is an extremely rare and newly recognised tumour. Here, we describe five cases of RH, one discovered by CT in an accident and the other four detected during routine examinations.

**Methods:**

Five cases of renal hemangioblastoma retrieved from the Department of Pathology, Fudan University Shanghai Cancer Center were studied and the literatures were reviewed. Immunohistochemistry was used to differentiate and confirm this tumour.

**Results:**

Pathological examination following tumour resection revealed RH in all cases, the first patient was also diagnosed with renal cell carcinoma (RCC), suggesting the possibility of VHL syndrome, but PCR sequencing analysis of the VHL gene confirmed no mutation in any of the three exons, implying sporadic disease .Histologically, the tumours were circumscribed, composed of sheets of oval or polygonal cells and a prominent vascular network. Tumour cells had pleomorphic nuclei, but mitotic figures were rare. The diagnosis of hemangioblastoma was confirmed by immunohistochemistry.

**Conclusions:**

RH is very rare and is challenging to differentially diagnose. Distinguishing RCC and RH is difficult and each has a different prognosis, so differentiating between them is essential for avoiding over-diagnosis and unnecessary treatment.

## Introduction

Hemangioblastoma is a benign tumour of uncertain histogenesis characterised by the presence of stromal cells and a rich vascular component.^1^ Most cases, which generally arise from the central nervous system (CNS), are sporadic, while approximately 25% are associated with von Hippel-Lindau (VHL) disease, an autosomal dominant disorder associated with germline mutations in the VHL tumour suppressor gene located on the short arm of chromosome 3.^2^ ^3^

Hemangioblastoma has also been reported outside the CNS, for example in the peripheral nerves,^4–6^ soft tissue,^7^ ^8^ liver,^9^ ^10^ lung,^9^ pancreas,^11^ kidney,^11–15^ retroperitoneum,^16^ urinary bladder,^11^ popliteal fossa^7^ ^8^ and nasal skin.^6^ In this study, we describe five renal hemangioblastomas (RHs), all of which had features very similar to those of other malignancies such as renal cell carcinoma (RCC). We also describe the pathological and immunohistochemical features of this rare disease.

## Materials and methods

All cases were sent to one of the authors for consultation. H&E-stained sections (4 mm thickness) were re-examined to evaluate the tumour's histological features and immunohistochemistry was performed with an avidin-biotin-complex immunoperoxidase technique. Antibody details are given table 1. Appropriate positive and negative controls were used throughout.

**Table 1:** Panel of antibodies used in this study

Antigen	Clone	Dilution	Source
S100	Polyclonal	1:2000	Dako, Carpinteria, California, USA
Vimetin	V9	1:200	Dako
EMA	E29	1:500	Dako
HMB-45	HMB-45	1:50	Dako
CD34	QBEnd10	1:20	BioGenex, San Ramon, California, USA
CD31	JC/70A	1:50	Dako
PAX8	Polyclonal	1:800	Proteintech Group, Chicago, Illinois, USA
AE1/AE3	AE1/AE3	1:100	Dako
Inhibin	R1	1:50	Dako
NSE	BBS/NC/VI-H14	1:200	Dako
CK7	OV-TL 12 ⁄ 30	1:50	Dako
CK8	CAM5.2	1:10	Becton Dickinson, San Jose, California, USA
CK	AE1/AE3	1:100	Dako

NSE, neuron-specific enolase.

Clinical demographics and follow-up data were obtained from medical records and the referring physicians. The institutional review board for human studies of Fudan University Shanghai Cancer Center approved this retrospective study. Patient records or information were anonymised and de-identified prior to analysis. Written informed consent was provided by participants or their next-of-kin for their clinical records to be used in this study.

## Results

### Clinical characteristics of five cases

#### Case 1

A 30-year-old man was admitted to the hospital for examination after an accident and a CT scan revealed a mass with a different density within a right kidney lesion. The malignancy was subsequently imaged by MRI. Surgical resection of the tumour revealed a collision tumour comprising RCC and extraneural hemangioblastoma, among which RCC accounts for less than 5% of the total tumour volume. The mass involved the renal cortex and medulla and measured 3.2×2.5×1.4 cm. VHL disease was initially suspected. However, PCR sequencing analysis of the *VHL* gene confirmed no mutation in any of the three exons, suggesting sporadic disease. The patient died before the next follow-up.

#### Case 2

A CT scan during a routine examination revealed that a 57-year-old woman had a mass in her right kidney. A radical nephrectomy was performed as a malignant tumour was suspected. H&E slides confirmed the diagnosis of RCC. No surgery or chemotherapy was carried out. The patient is currently alive with no evidence of recurrent tumour or the development of other tumours.

#### Case 3

The patient was a 48-year-old man with no hereditary diseases. When he visited the hospital for evaluation of organ function, abdominal sonography revealed a tumour mass in the right kidney. A CT scan confirmed a 2.3 cm mass with heterogeneous density in the lower portion of the right kidney. As RCC was suspected, the patient underwent a right radical nephrectomy. There was no evidence of tumour recurrence 42 months after the surgery. Follow-up magnetic resonance brain imaging did not any reveal tumours.

#### Case 4

A 25-year-old man was confirmed to have a left renal tumour during a routine examination, and was admitted to hospital for urological examination. The patient was asymptomatic with a normal appetite, no abdominal pain, no weight changes, and no family history of renal disease. CT imaging revealed a 3.6 cm well-defined, round, heterogeneously enhancing mass in the left kidney. Nephrectomy was performed and a 4.1 cm encapsulated tumour removed. Follow-up at 27 months confirmed no tumour recurrence or metastasis.

#### Case 5

A previously healthy 36-year-old woman presented with a tumour on the left kidney during a routine examination. The tumour was an unencapsulated but sharply circumscribed nodule. Clear cell RCC (CCRCC) was diagnosed. Immunohistochemistry of a tissue sample suggested a diagnosis of RH. The patient was alive 3 months later.

### Microscopic features

**Figure 1:**
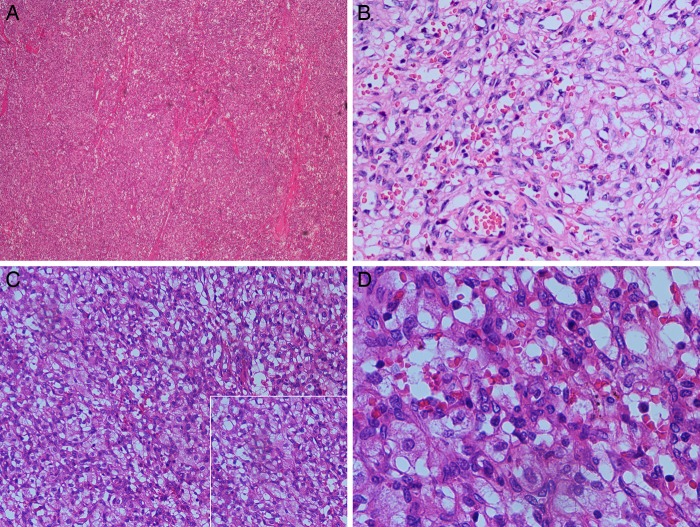
Histological features. (A) Most tumours were solid and were traversed by arborising thin-walled blood vessels. (B) Tumours were composed of plump spindle cells with palely eosinophilic cytoplasm. (C) The microvacuolated cells often mimicked lipoblasts or renal cell carcinoma and were mildly atypical. (D) Abundant anastomosing thin blood vessels surrounding bland-appearing stromal cells. Under a high-power field, scattered tumour cells have highly pleomorphic nuclei.

**Figure 2:**
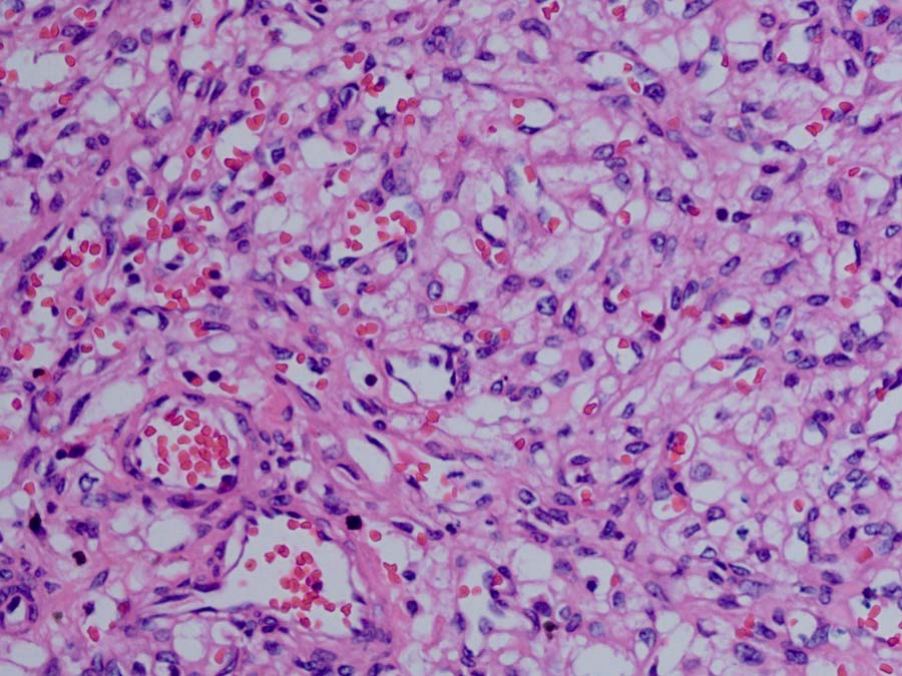
Blood vessels often had ectasia or pericytomatous configurations.

Microscopically, most tumours were well circumscribed and well demarcated from the surrounding renal parenchyma; only one tumour had areas of poorly marginated growth. The RHs all consisted of sheets of oval or polygonal cells traversed by a prominent, arborising vascular network (figure 1A, B). The tumours also included small microvacuolated cells with palely eosinophilic or clear cytoplasm (figure 1C, D). These microvacuolated cells often mimicked RCC. All tumours had a complex capillary network and blood vessels, which were thin-walled and lined with flat to plump endothelial cells, often had ectasia or pericytomatous configurations (figure 2). Case 1 had a focally marked nuclear pleomorphism; nuclear pleomorphism of the other cases were generally difficult to detect. There were a very few mitotic figures in one case and none at all in the other cases. In all cases, there were minimal areas of stromal hyalinisation. Necrosis or lymphovascular invasion was not identified in any case.

### Immunohistochemical findings

**Figure 3:**
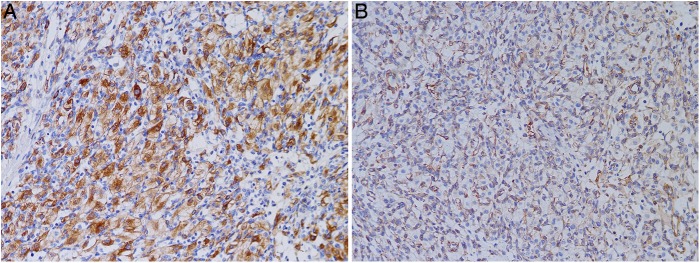
In most cases, tumour cells expressed inhibin (A) and vimentin (B).

**Figure 4:**
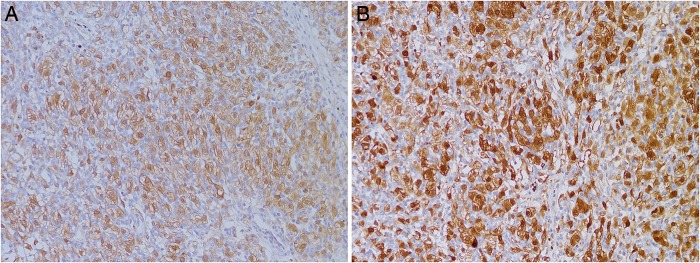
Tumour cells were positive for neuron-specific enolase (A) and S100 protein (B).

**Figure 5:**
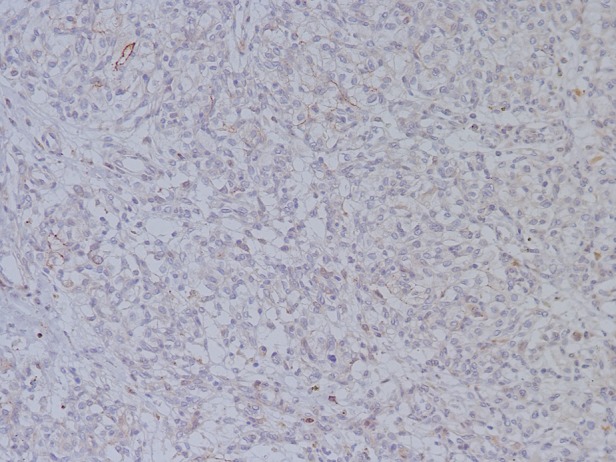
EMA was focally positive in stromal cells in some cases.

**Figure 6:**
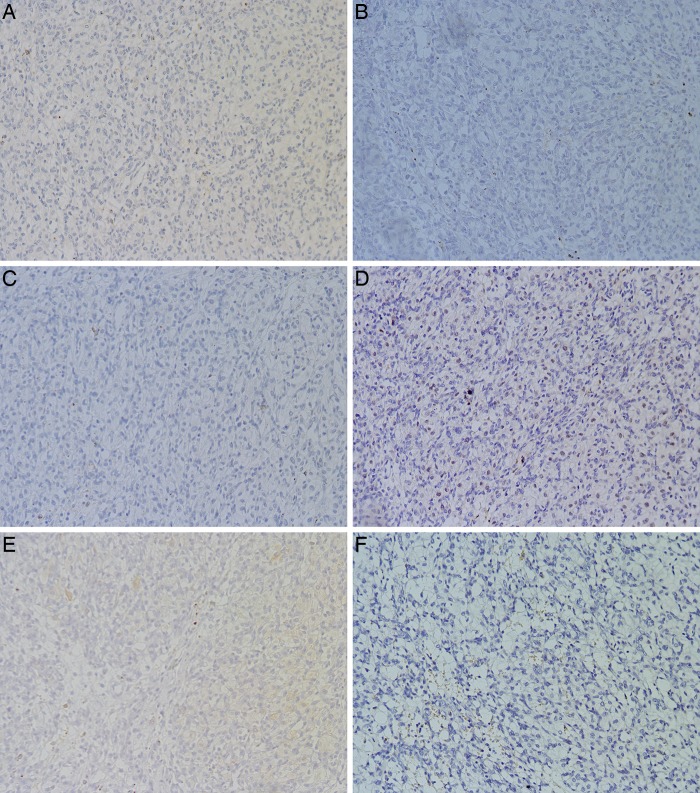
Most tumours except case 1 were negative for CK (A), CK7 (B), CK8 (C), PAX8 (D), AE1/AE3 (E) and HMB45 (F).

**Figure 7:**
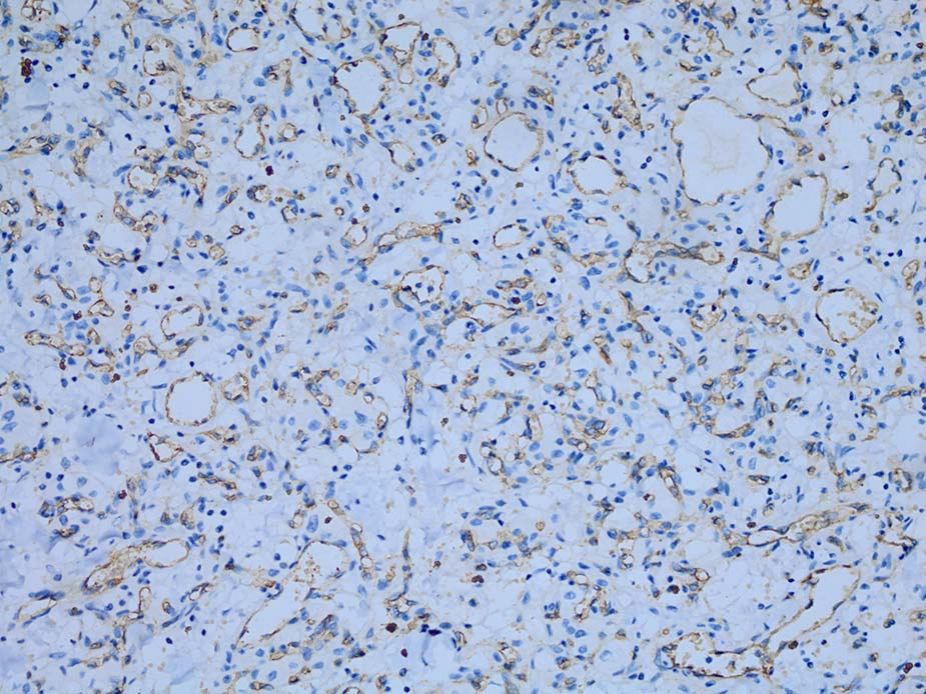
CD34 stains highlighted the capillary network but not the tumour cells.

Most cases had similar immunohistochemical profiles. Tumour cells expressed inhibin (3/5) and vimentin (3/5) in 60% of cases (figure 3A, B). Neuron-specific enolase (NSE) and S100 protein were expressed in all cases (figure 4A, B). EMA was focally positive in stromal cells in half of the cases (figure 5). Four of the tumours were negative for CK, CK7, CK8, PAX8, AE1/AE3 and HMB45 (figure 6A–F). CD31 or CD34 stains highlighted the capillary network but not tumour cells (figure 7). Interestingly, one case had markers for both hemangioblastoma and CCRCC, suggesting the possibility of VHL syndrome. Immunohistochemistry data are summarised in table 2.

**Table 2:** Results of immunohistochemical staining in renal hemangioblastoma (RH)

	Case 1					
	RH	RCC	Case 2	Case 3	Case 4	Case 5
Vimentin	+	+	+	−	−	+
S100	+	−	+	+	+	+
Inhibin	+	−	−	+	−	+
NSE	+	+/−	N/A	+	+	+
EMA	+	+	−	−	N/A	+
CK7	−	+	−	−	−	−
CK8	−	+	−	−	−	−
CK	N/A	N/A	−	−	N/A	−
CD10	−	+	−	−	−	+
AE1/AE3	−	+	−	−	−	−
HMB45	N/A	N/A	N/A	−	−	−
CD31	N/A	N/A	+	N/A	N/A	+
CD34	+	−	+	+	+	+
PAX8	−	+	−	−	−	−

NA, stain not performed; NSE, neuron-specific enolase; RCC, renal cell carcinoma; +, positive; −, negative; +/−, variable.

## Discussion

Hemangioblastoma is a benign tumour of uncertain histogenesis that generally occurs in a relatively restricted area of the CNS.^17^ Extraneural cases are seldom seen and usually accompany VHL disease.^13^ This report describing two female and three male patients (median age 39.2 years; range 25–57 years) with RH adds to the total number of published cases (table 3). In contrast to CNS hemangioblastoma which is predominantly found in males, renal tumours affect both male and female patients.^1^ ^18^ CNS hemangioblastoma is found predominantly in the third to the fifth decades of life. However, patients in our series were younger than those in previous reports, but this may not be significant as our sample size was small.

**Table 3:** Reported cases of renal hemangioblastoma

References	Age	Gender	Presence of VHL	Outcome
Nonaka *et al*^32^	71	F	Not stated	No recurrence 9 years after nephrectomy
Ip *et al*^13^	58	M	No	No recurrence 2 years after nephrectomy
Ip *et al*^13^	55	F	No	Well after nephrectomy
Wang *et al*^15^	29	M	No	No recurrence 20 months after nephrectomy
Verine *et al*^33^	64	M	No	No recurrence 1 year after nephrectomy
Wang *et al*^14^	61	M	No	No recurrence 1 year after nephrectomy
Liu *et al*^36^	36	F	No	No recurrence 1 year after nephrectomy
Current case 1	30	M	Suggestive of VHL	Died after nephrectomy
Current case 2	48	M	No	No recurrence 3.5 years after nephrectomy
Current case 3	25	M	No	No recurrence 27 months after nephrectomy
Current case 4	36	F	No	Well after nephrectomy
Current case 5	57	F	No	No recurrence 5 months after nephrectomy

VHL, von Hippel-Lindau disease.

VHL disease is an autosomal dominant hereditary disorder characterised by retinal and CNS hemangioblastomas, pheochromocytoma and CCRCC.^19–21^ Mutations in the VHL gene lead to the development of several benign or malignant tumours, and cysts in many organ systems. The VHL gene which is located on chromosome 3p25, encodes for a 213 amino acid tumour suppressor protein that plays a key role in the regulation of the hypoxia response pathway.^2^ ^22^ Aberrations in VHL function, either through mutation or other mechanisms, lead to the accumulation of hypoxia-inducible factor, which then transcriptionally upregulates a sequence of hypoxia responsive genes, including epidermal growth factor, vascular endothelial growth factor, platelet-derived growth factor and other pro-angiogenic factors, resulting in tumour formation.^23^ It is currently recommended that all patients with CNS hemangioblastoma undergo testing for VHL germline mutations. According to the WHO Blue Book, the clinical diagnosis of VHL disease is based on the presence of hemangioblastoma in the CNS or retina, the presence of one of the typical VHL-associated tumours, or a previous family history.^1^ In the present study, one case which expressed markers of hemangioblastoma and CCRCC was subjected to Sanger DNA sequencing. Each exon was identified and confirmed by both forward and reverse directional analyses but no mutation was detected. None of the other patients met any of these criteria, and are considered to have sporadic disease.

The tumours in these five cases were morphologically similar to lesions arising in the CNS, which are also composed of sheets of oval or polygonal cells with palely eosinophilic and clear microvacuolated cytoplasm, separated by a delicate capillary network, and usually with interspersed larger thin-walled and thick-walled blood vessels.

Compared with CNS hemangioblastoma, the results of immunohistochemical staining reported by many authors do not suggest a specific line of differentiation for RH tumours.^16^ ^24–27^ Markers currently used for these lesions include epithelial (cytokeratins), muscle (MSA, desmin, calponin), neural (S100 protein, neuron specific enolase) and other generic mesenchymal markers (vimentin); vimentin, S100 protein, NSE and inhibin are reported to express constantly in this tumour.

In the current study, S100 and NSE appeared to be the most sensitive markers and were expressed in all cases, as was inhibin. Vimentin expression was observed in 60% of cases. Of note, expression of inhibin was less in our cases than reported in the RH literature; sample limitations may explain this discrepancy. The fact that vimentin is strongly expressed in these tumours has led investigators to suggest they have an undifferentiated mesenchymal origin.^4^ Expression of GLUT1 in CNS hemangioblastoma has been reported to be helpful for differential diagnosis with metastatic RCC, as is the strong endothelial staining observed with most hemangioblastomas, unlike RCC, which does not show such a staining pattern.^28^ However, this finding is controversial, as some authors^29^ doubt the low specificity and tendency for high background staining. Recently, expression of brachyury has been described in cerebellar hemangioblastoma tumour cells.^30^ ^31^ Tirabosco's group^30^ reported nuclear expression of brachyury in stromal cells in all 14 CNS cases they examined. However, Doyle and Fletcher^29^ reported that brachyury was not expressed in extraneural tumours in their series.

RH is frequently misdiagnosed as RCC, and is likely to be under-recognised as it mimics many tumour types morphologically and is usually not considered in a differential diagnosis.^32^ Correctly diagnosing RH is challenging and important because sporadic RH does not require further treatment and the prognosis is much better than that of malignant RCC.^14^ ^15^ The presence of pericytomatous growth patterns and intracytoplasmic lipid vacuoles strongly suggests hemangioblastoma,^13^ ^33^ although both tumour types have similar morphological features, such as clear cytoplasm and a vascular network.^34^ Immunohistochemistry is useful for differentiating morphologically similar neoplasms. RCC is usually positive for cytokeratin (predominantly low molecular weight), PAX8 and AE1/AE3.^27^ ^34^ However, the immunohistochemistry data for cytokeratin, PAX8, and AE1/AE3 ruled out the possibility of RCC in our study. In contrast to hemangioblastoma, RCC is usually negative for inhibin, S100 and NSE. Other differential diagnoses include adrenal cortical carcinoma, epithelioid angiomyolipoma and paraganglioma,^35^ which can offer critical diagnostic information.

**Take home messages**

- Extraneural renal hemangioblastoma (RH) is a rare, newly recognised tumour with morphological features similar to its cerebellar counterpart.
- This report describes the morphological features and immunohistochemical characteristics of five cases of RH and summarises the findings.
- RH is frequently misdiagnosed, so immunohistochemistry is essential for differentiating RH from morphologically similar neoplasms, such as renal cell carcinoma.
